# Beliefs about chelation among thalassemia patients

**DOI:** 10.1186/1477-7525-10-148

**Published:** 2012-12-07

**Authors:** Felicia L Trachtenberg, Lauren Mednick, Janet L Kwiatkowski, Ellis J Neufeld, Dru Haines, Zahra Pakbaz, Alexis A Thompson, Charles T Quinn, Robert Grady, Amy Sobota, Nancy Olivieri, Robert Horne, Robert Yamashita

**Affiliations:** 1 New England Research Institutes, 9 Galen Street, Watertown, MA, 02472, USA; 2 Boston Children’s Hospital, 300 Longwood Ave, Boston, MA, 02115, USA; 3 Children's Hospital of Philadelphia, Division of Hematology, 34th Street and Civic Center Boulevard, Colket Translational Research Building, Room 11024, Philadelphia, PA, 19104, USA; 4 Children’s Hospital & Research Center Oakland, Hematology/Oncology Department, 747 52nd Street, Oakland, CA, 94609, USA; 5 Children’s Memorial Hospital, 2300 Children’s Plaza, Box #30, Chicago, IL, 60614, USA; 6 University of Texas Southwestern Medical Center, Cincinnati Children’s Hospital Medical Center, 3333 Burnet Ave, Cincinnati, OH, 45229, USA; 7 Weill Cornell Medical College University, 525 E 68th Street, Box 103, New York, NY, 10065, USA; 8 Toronto General Hospital, University Health Network, 200 Elizabeth Street, Eaton Wing North, 10th Floor, Room 225, Toronto, Ontario, M5G 2C4, Canada; 9 University of London, School of Pharmacy, Mezzanine Floor, BMA House, Tavistock Square, London, WC1H 9JP, UK; 10 Liberal Studies Department, California State University San Marcos, 333 S. Twin Oaks Valley Road, San Marcos, CA, 92096-0001, USA

**Keywords:** Thalassemia, Necessity, Concerns, Chelation, Adherence

## Abstract

**Background:**

Understanding patients’ views about medication is crucial to maximize adherence. Thalassemia is a congenital blood disorder requiring chronic blood transfusions and daily iron chelation therapy.

**Methods:**

The Beliefs in Medicine Questionnaire (BMQ) was used to assess beliefs in chelation in thalassemia patients from North America and London in the Thalassemia Longitudinal Cohort (TLC) of the Thalassemia Clinical Research Network (TCRN). Chelation adherence was based on patient report of doses administered out of those prescribed in the last four weeks.

**Results:**

Of 371 patients (ages 5-58y, mean 24y), 93% were transfused and 92% receiving chelation (26% deferoxamine (DFO; a slow subcutaneous infusion via portable pump), 63% oral, 11% combination). Patients expressed high “necessity” for transfusion (96%), DFO chelation (92%) and oral chelation (89%), with lower “concern” about treatment (48%, 39%, 19% respectively). Concern about oral chelation was significantly lower than that of DFO (p<0.001). Self-reported adherence to chelation was not associated with views about necessity or concerns, but negatively correlated with perceived sensitivity to DFO (Sensitive Soma scale; r=−0.23, p=0.01) and side effects of oral chelation (r=−0.14, p=0.04). High ferritin iron levels, potentially indicating lower adherence, were found in 41% of patients reporting low necessity of oral chelation compared to 24% reporting high necessity (p=0.048). Concerns about treatment were associated with lower quality of life and more symptoms of anxiety and depression.

**Conclusions:**

Despite their requirement for multimodal therapy, thalassemia patients have positive views about medicine, more so than in other disease populations. Patients may benefit from education about the tolerability of chelation and strategies to effectively cope with side effects, both of which might be beneficial in lowering body iron burden.

**Clinicaltrials.gov identifier:**

NCT00661804

## Background

Thalassemia is a congenital blood disorder that is often managed with chronic blood transfusions, typically every 2–4 weeks, which leads to progressive iron overload. Daily iron chelation therapy is prescribed to manage the transfusional iron overload and attempt to prevent progressive organ failure (heart, endocrine, liver). Life expectancy is directly related to the quality of chelation therapy, and poor adherence to treatment increases the risk of complications and shortens survival. Patient adherence practices are generally seen as a singular action, a one-dimensional phenomenon where the patient simply chooses not to do their chelation therapy. In this model, either simpler chelation methods and/or patient education were the defined solutions to failed practice. Until recently, parenteral treatment with nearly daily prolonged infusion of deferoxamine was the only available treatment, However, despite the development of new oral chelators and the development of patient education interventions, patient adherence continues to be a problem in thalassemia. Developing effective adherence interventions requires better analytic models of adherence practice.

Patients’ concerns about their treatments and their beliefs in its necessity appear to be critical in understanding patient adherence practices. The Beliefs about Medicines Questionnaire (BMQ) was designed to examine this and has been used in many disease populations, including asthma, diabetes, renal disease, cardiac disease, oncology, hypertension, arthritis, bowel disease, HIV, migraines, depression, and psychiatric disorders [1-15]. A second instrument, the Sensitive Soma Assessment Scale (SSAS) is another instrument that is designed to assess a patient’s perceived sensitivity to medicines, and this preconception is likely related to the report of side-effects [16].

The BMQ scales have been found to correlate with adherence to treatment across many diseases and populations [1-14,17,18]. Beliefs that treatment is less necessary or feelings of greater concerns about treatment are associated with lower adherence to that treatment. The necessity-concerns difference score has been found to be especially related to adherence, suggesting that “medication beliefs were more powerful predictors of reported adherence than the clinical and sociodemographic factors” [2]. The SSAS is a new instrument that early use in AIDS, hypertension, travel clinic, and undergraduate students suggests has a strong predictive association with adherence [16]. Given the importance of adherence to thalassemia treatment, understanding how patients’ beliefs in chelation therapy relate to their adherence (or lack thereof) may help providers better assist their patients. This is especially important because chelation therapy has two main forms: subcutaneous slow infusions by a portable pump (deferoxamine) and oral (deferasirox, deferiprone).

The Thalassemia Clinical Research Network (TCRN) is an NIH/NHLBI-funded network composed of five core thalassemia centers in North America, one in London, and their associated satellite sites. In May 2007, the TCRN launched the Thalassemia Longitudinal Cohort (TLC) study with baseline and annual collection of patient questionnaires, clinical history, results of standard-of-care procedures, and data regarding treatments for thalassemia and its complications. The aim of the present study is to summarize Beliefs in Medicine Questionnaire (BMQ) results for patients with thalassemia, compare beliefs in deferoxamine (DFO) vs. oral chelators, and assess effect on quality of life, anxiety, depression, and chelation adherence. This unique large international study of thalassemia patients spanning a wide age range (5–58 years) is the first to examine the medical beliefs of patients with thalassemia. Given the thalassemia literature which has emphasized the need for better education to improve adherence, we hypothesized that patients who viewed chelation as high necessity would show better adherence, and patients with high concern would show poorer adherence.

## Methods

The TCRN TLC protocol was approved by the TCRN Data and Safety Monitoring Board and by the ethical review boards of all TCRN institutions. Informed consent was obtained for all participants. Eligibility for the TLC study included patients with all thalassemia syndromes who required at least annual monitoring for end-organ injury related to thalassemia. 428 patients were enrolled in the TLC from 2007–2009. Patients with a prior successful hematopoietic stem cell transplant (N=12) were excluded from this analysis. 371 patients completed the BMQ at baseline and were included in this analysis.

At baseline and annually, demographic information was collected and participants (age 14+) and/or their parents (age <16y) asked to complete the BMQ (Appendix 1). Participants were surveyed about their general beliefs in medicine, and also those specific to transfusion and chelation. The BMQ is a validated instrument [1,19] containing 5 scales (Appendix 1), each with 4–5 items: (1) General-Overuse, (2) General-Harm, (3) General-Benefit, (4) Specific-Necessity, and (5) Specific-Concerns. The first three relate to general beliefs in medicine: (1) the notion that doctors tend to overuse and trust medicines too much, (2) the potential for medicines to be harmful, addictive, and poisonous, and (3) the potential for medicines to be helpful and allow for longer better lives. The last two scales (Specific-Necessity, Specific-Concerns) apply to the particular medicine the person is taking and measure how necessary the patient feels their medicine is for them (necessity), and perceptions of the potential negative consequences of the medication such as long-term effects, dependence, and disruptiveness (concerns). Higher necessity scores indicate higher beliefs that transfusion/chelation is necessary for their health. Higher concern scores indicate higher levels of concern about treatment. Participants not on transfusion or chelation therapy completed only the general scales. Participants on chelation were surveyed about the specific chelator they were using. Participants were using a range of chelators, including deferoxamine (DFO), deferasirox, deferiprone (available only for compassionate use in North America), and combination therapy. Patients on combination chelation (both DFO and an oral chelator) completed the scales twice, once for each chelator. The same survey also included the Sensitive Soma scale [1,16], 5 items that assess perceptions of personal sensitivity to the potential adverse effects of medication (Appendix 1). In addition, a single item from the original BMQ item pool [1] was added asking how well patients can cope without transfusion/chelation. Finally, one additional item was added asking about unpleasant side effects of transfusion/chelation. All questions were asked on a 5-point Likert scale of strongly disagree to strongly agree (Appendix 1).

Participants aged 14 and older were asked to complete the hospital anxiety and depression scale (HADS) [20], as well as the SF-36v2 health survey [21] which measures health-related quality of life (QOL) in 8 subscales and two summary scores: physical component and mental component. Parents of children <14 years at baseline were asked to complete the PF-28 child health questionnaire (CHQ) [22], which measures quality of life (QOL) in children in 12 subscales and two summary scores (physical and mental). Participants or their parents were surveyed about their chelation use (number of doses taken in the past week and month), and study coordinators recorded the prescribed chelator dosage. Chelation adherence was defined as percent of doses administered in the last four weeks (patient report) out of those prescribed (coordinator chart review). Finally, serum ferritin, liver iron concentration (LIC) by FerriScan®, MRI, SQUID, or liver biopsy, and clinical complications were recorded from chart review. Ferritin ≥2500 ng/ml and LIC ≥15 mg Fe/g dry were considered markers of inadequate chelation. Potential complications included cardiac complications (congestive heart failure, ventricular arrhythmia, low cardiac T2*<12 ms by MRI), endocrine complications (diabetes type I, diabetes type II, growth hormone deficiency, hypothyroidism, hypoparathyroidism, hypogonadotropic hypogonadism), liver disease (cirrhosis), and transfusion related complications and infections (alloimmunization, active hepatitis C, chronic active hepatitis B, HIV).

### Statistical analysis

This manuscript reports baseline data from this ongoing study, except for the analysis of changes over time and the analysis of forms completed by both child and parent in the same family, which included all available follow-up. Statistical analyses were performed with SAS 9.2 for Windows (SAS Institute Inc., Cary, NC, USA), and in all analyses p<0.05 was considered statistically significant. BMQ scales were scored according to the BMQ scoring instructions (Appendix 1) and internal consistency measured with Cronbach’s alpha. Consistent with the development of the scales [2], high necessity and concerns were defined as scores above the midpoint, and four groups were defined: accepting (high necessity, low concerns), ambivalent (high necessity, high concerns), indifferent (low necessity, low concerns), and skeptical (low necessity, high concerns) [11]. The necessity-concerns difference score was calculated and those with a negative difference score were considered an important subset [2].

To test for differences in beliefs between children and their parents (N=30 pairs), paired t-tests were used. To test for differences in beliefs regarding transfusion vs. chelation and DFO vs. oral chelation, t-tests were used; participants on combination chelation (both DFO and oral) were excluded from analysis of DFO vs. oral chelation. A multivariate analysis of covariance model with backwards elimination was used to model predictors of beliefs about medicine. Potential predictors included respondent (parent vs. self), age group of the patient (child 5–17 years, young adult 18–24, adult 25+), oral chelator (deferasirox vs. deferiprone), monotherapy vs. combination chelation (DFO, oral, combination), gender, race (White, Asian, other), country (US, Canada, UK), diagnosis (β vs. E-β thalassemia), regular transfusion (at least 8 transfusions in the past year), and number of clinical complications.

Correlations between BMQ scales were calculated, as well as correlations with QOL summary scales, HADS scales (anxiety, depression), self-reported adherence, and average ferritin and LIC in the past year (both log-transformed due to skew). Correlations with adherence, ferritin, and LIC were also calculated in the subset of adults (18+), as children are often not in control of their adherence. Analysis of variance (ANOVA) was used to compare adherence rates by high/low necessity and concerns, as well as negative/non-negative necessity concerns difference. Chi-square and Fisher exact tests examined associations between high/low necessity and concerns with high ferritin (≥2500 ng/ml) and LIC (≥15 mg Fe/g dry). To assess changes in BMQ responses over time, change in each scale from baseline was computed. Repeated measures models were used to test for linear changes over follow-up. Finally, t-tests were used to test for changes over time in individuals who changed chelators during follow-up (DFO to oral or vice versa).

## Results

At baseline, 371 TLC participants/parents completed the BMQ. Surveys were completed by 231 adult participants, 104 parents/guardians (mean age 10.2y, range 5–16) and 49 children (mean age 14.9y, range 10–17), including 30 pairs with forms completed by both child and parent (13 at baseline and 17 at annual follow-up, all ages 14–15). Most participants were on transfusion (93%) and chelation (92%) therapy, with 24% chelating with DFO, 58% on an oral chelator, and 10% on combination therapy (Table 1). Patients from London constituted 53% of TLC participants on deferiprone monotherapy and 27% of those on deferiprone combination chelation. The participants ranged in age from 5–58 years, with an average of 24 years. Approximately 75% of respondents had beta thalassemia major (transfused at least 8 times in the past year). Participants had an average of 1.3 clinical complications (median 1, range 0–8, 21% with >2 complications). Internal consistency (Cronbach’s alpha) for all BMQ scales was within the range reported in scale development [1,16]: 0.80-0.84 for the Specific-Necessity scales, 0.68-0.73 for the Specific-Concerns scales, 0.63-0.71 for the general scales, and 0.84 for Sensitive Soma.

**Table 1:** Demographics for the Thalassemia Longitudinal Cohort (TLC) participants completing the Beliefs in Medicines Questionnaire (BMQ) (N=371)^a^

	
Age (years), mean (SD), range	24.0 (12.6), 5.0 - 58.3
Gender, N (%)	
Male	176 (47.4%)
Female	195 (52.6%)
Race, N (%)	
White	181 (49.6%)
Asian	163 (44.7%)
Other	21 (5.8%)
Country, N (%)	
US	269 (72.5%)
Canada	64 (17.3%)
UK	38 (10.2%)
Thalassemia diagnosis, N (%)	
β-thal transfused 8+ times	279 (75.2%)
β-thal transfused <8 times	28 (7.6%)
β-thal not transfused	4 (1.1%)
E-β-thal transfused 8+ times	32 (8.6%)
E-β-thal transfused <8	8 (2.2%)
E-β-thal not transfused	1 (0.3%)
HbH	6 (1.6%)
HbH Constant Spring	8 (2.2%)
Alpha-thalassemia	5 (1.4%)
Chelation, N (%)	
None	30 (8.1%)
Deferoxamine (DFO)	89 (24.0%)
Deferasirox	200 (53.9%)
Deferiprone	15 (4.0%)
Deferoxamine + Deferasirox	11 (3.0%)
Deferoxamine + Deferiprone	26 (7.0%)
Serum ferritin (ng/ml), median (range) ^b^	1296.8 (75.0 - 18453.5)
Liver iron concentration (LIC in mg Fe/g dry), median (range) ^b^	8.2 (0.4 – 67.9)

### BMQ scales for transfusion and chelation

Of those on transfusion therapy, 96% reported high necessity (> midpoint of 15 points), with 48% reporting high concerns. For chelation therapy, high necessity was reported in 92% of those on DFO and 89% of those on oral chelation, with concerns reported in 39% and 19% respectively. Participants rated significantly higher the necessity of transfusion compared to chelation (paired t-test, p<0.001 for both DFO and oral chelation), with no specific items responsible for this difference and no significant difference between transfusion and DFO chelation on the Specific-Concerns scale. There were no significant differences found on reported ability to cope without transfusion, DFO, or oral chelation. However, greater side effects were reported for DFO compared to transfusion and oral chelation (p≤0.001 for both).

There was no significant difference between chelators on the Specific-Necessity scale, but participants expressed higher concern about chelation with DFO compared to oral chelation (p<0.001, Table 2), with an increase of 1.74 ± 0.48 points largely stemming from responses to the item on “disruption to life”. Participants on DFO compared to oral chelation perceived their bodies to be more sensitive to medications (Sensitive Soma scale 12.9 vs. 11.1, p<0.001). There were no differences in beliefs between patients on deferasirox vs. deferiprone. However, patients on combination therapy reported lower beliefs in the necessity of each chelator compared to patients on monotherapy (p≤0.002 for both DFO and oral chelation, Table 3).

**Table 2:** Beliefs in Medicines Questionnaire (BMQ) scale responses by age group^a^ and respondent

**BMQ Scale**		**N^a^**	**Mean (SD)**			
			**Child**	**Young Adult**	**Adult**	**Parent of Child**
			**Age 10–17 N=49**	**Age 18–24 N=75**	**Age 25+ N=156**	**Age 5–16 N=104**
Specific Necessity–DFO^b^		132	21.0 (3.3)	20.5 (3.4)	21.6 (3.7)	21.3 (3.1)
Specific Concerns–DFO^c^		131	14.4 (5.0)	12.1 (4.2)	12.9 (3.5)	16.7 (2.8)
Specific Necessity-Oral Chelator^b^		256	19.0 (3.9)	20.2 (3.9)	20.9 (3.8)	21.4 (3.4)
Specific Concerns-Oral Chelator^c^		256	10.8 (3.6)	11.8 (3.9)	12.2 (3.6)	13.0 (4.0)
General Overuse^d^		375	11.6 (2.5)	11.8 (2.8)	11.8 (2.8)	12.2 (2.9)
General Harm^e^		380	9.5 (2.4)	9.3 (2.7)	8.9 (2.8)	9.6 (3.2)
General Benefit^f^		378	15.6 (2.3)	15.7 (2.1)	15.9 (2.4)	16.3 (2.2)
Sensitive Soma^g^		379	11.3 (3.9)	11.4 (3.8)	12.0 (3.9)	11.7 (3.7)
**Comparison of DFO vs. Oral Chelation across age groups**						
BMQ Scale	Specific Necessity	Specific Concerns	General Overuse	General Harm	General Benefit	Sensitive Soma
p-value^h^	0.30	**<0.001**	0.08	0.20	0.08	**<0.001**

^a^ includes 13 parent/child pairs of responses from the same family.

^b^ 5 items measuring how necessary participants perceive chelation to be. Higher scores indicate higher perceived necessity (0–25 points). Participants on both DFO and oral chelation responded separately for each chelator.

^c^ 5 items measuring participants’ concerns with chelation. Higher scores indicate higher levels of concern (0–25 points). Participants on both DFO and oral chelation responded separately for each chelator.

^d^ 4 items measuring beliefs that doctors tend to overuse and trust medicines too much. Higher scores indicate higher levels of this belief in the overuse of medicines (0–20 points).

^e^ 4 items measuring beliefs that medicines tend to be harmful, addictive, and poisonous. Higher scores indicate higher levels of this belief in the harm of medicines (0–20 points).

^f^ 4 items measuring beliefs that medicines are helpful and make people live longer better lives. Higher scores indicate higher levels of this belief in the benefit of medicines (0–20 points).

^g^ 5 items measuring perceptions of personal sensitivity to the potential adverse effects of medication. Higher scores indicate higher levels of this perception of sensitivity to medicines (0–25 points).

^h^ Across all ages and respondents, t-test for DFO vs. oral chelation, excluding participants on both. Participants on DFO compared to oral chelation report belief in slightly higher General Overuse (12.4 vs. 11.8) and General Harm (9.4 vs. 9.0), slightly lower General Benefit (15.6 vs. 16.1), and significantly higher Sensitive Soma (12.9 vs. 11.1).

**Table 3:** Predictors of Beliefs in Medicines Questionnaire (BMQ) scale responses^a^

	**Slope (SE)**	**p-value**
**Predictors of Specific Necessity - DFO**		
Monotherapy vs. combination therapy	2.39 (0.75)	0.002
Regularly transfused	2.84 (1.02)	0.006
Number of complications	0.39 (0.18)	0.031
**Predictors of Specific Concerns - DFO**		
Age group		0.04
Young adult vs. child	−2.45 (0.95)	
Adult vs. child	−1.87 (0.99)	
Race		<0.001
Asian vs. white	1.91 (0.66)	
Other vs. white	−5.25 (2.15)	
Country		0.004
Canada vs. US	1.89 (0.93)	
UK vs. US	2.80 (1.03)	
**Predictors of Specific Necessity – Oral Chelator**		
Monotherapy vs. combination therapy	3.47 (0.66)	<0.001
Parent vs. self	1.06 (0.53)	0.048
Race		<0.001
Asian vs. white	−0.19 (0.46)	
Other vs. white	−3.74 (1.01)	
Country		0.002
Canada vs. US	0.31 (0.68)	
UK vs. US	2.49 (0.72)	
**Predictors of Specific Concerns - Oral Chelator**		
Parent vs. self	2.61 (0.77)	0.001
Age group		0.005
Young adult vs. child	1.42 (0.80)	
Adult vs. child	2.39 (0.74)	
Race		<0.001
Asian vs. white	1.63 (0.49)	
Other vs. white	2.94 (1.01)	
Female vs. male	−1.19 (0.46)	0.01
**Predictors of General Overuse**		
Race		0.006
Asian vs. white	0.26 (0.30)	
Other vs. white	2.01 (0.62)	
Country		0.05
Canada vs. US	0.90 (0.37)	
UK vs. US	0.48 (0.48)	
**Predictors of General Harm**		
Age group		0.04
Young adult vs. child	−0.20 (0.40)	
Adult vs. child	−0.91 (0.37)	
Race		0.02
Asian vs. white	0.19 (0.31)	
Other vs. white	1.76 (0.64)	
Country		0.007
Canada vs. US	0.03 (0.39)	
UK vs. US	1.64 (0.52)	
**Predictors of General Benefit**		
Parent vs. self	0.62 (0.27)	0.02
Race		0.03
Asian vs. white	0.08 (0.25)	
Other vs. white	−1.33 (0.53)	
**Predictors of Sensitive Soma**		
Number of complications	0.29 (0.12)	0.02

^a^ Multivariate analysis of covariance model with backwards elimination. Predictors: respondent (parent vs. self), age group (child 5–17, young adult 18–24, adult 25+), gender, race (White, Asian, other), country (US, Canada, UK), diagnosis (β vs. E-β thal), regularly transfused (at least 8 transfusions in the past year), and number of complications (congestive heart failure, ventricular arrhythmia, low cardiac T2* by MRI, type I or II diabetes, growth hormone deficiency, hypothyroidism, hypoparathyroidism, hypogonadotropic hypogonadism, cirrhosis, alloimmunization, active hepatitis C, chronic active hepatitis B, and HIV).

Few participants had higher concerns than necessity (0.9% for transfusion and 6% for each type of chelator). Views about DFO and oral chelation, respectively, were 56% and 73% accepting (high necessity, low concerns), 36% and 16% ambivalent (high necessity, high concerns), 5% and 8% indifferent (low necessity, low concerns), and 3% and 4% skeptical (low necessity, high concerns).

### Predictors of BMQ scales

Parents expressed significantly higher levels of necessity and concern about oral chelation compared to children, as well as higher beliefs in the general benefit of medicines (Tables 2, 3). The largest difference on a specific item was worrying about long-term effects (3.40 vs. 4.26 for DFO; 2.59 vs. 3.47 for oral chelation). In analysis of the 30 pairs of parent/child reporters, all children aged 14-15y, parents reported significantly higher levels of concern about oral chelation; there was a similar trend for necessity of oral chelation and general benefit, which was non-significant, possibly due to small sample size. Many of the BMQ scale scores also increased significantly with age (Table 3). Belief in the necessity of oral chelation was higher in adults and young adults compared to children. Concerns about oral chelation were higher in adults aged 25+ compared to children. On the other hand, concerns about DFO were higher in children compared to adults and young adults. In addition, adults aged 25+ scored lower on the General-Harm scale compared to young adults and children, indicating lower belief that medicines are harmful.

Predictors of beliefs in medicine responses are summarized in Table 3. There were few gender differences on BMQ scales, but many racial differences. Asians participants reported higher levels of concern about chelation compared to White participants. Participants of other races reported lower levels of concern about DFO, but higher levels of concern and lower levels of necessity for oral chelation compared to White participants. Participants of other races also scored higher on the General-Overuse and General-Harm scales, and lower on the General-Benefit scale, compared to White participants. Furthermore, there were several differences in BMQ scores between participants in different countries. American participants reported lower levels of concern about DFO compared to those in Canada and the UK. Canadians scored higher on the General-Overuse scale compared to Americans. Additionally, participants in the UK scored higher on the General-Harm scale, but higher on the Specific-Necessity of oral chelation compared to Americans and Canadians. Regularly transfused patients reported a higher necessity of DFO chelation. Higher numbers of clinical complications were associated with higher necessity of DFO and higher Sensitive Soma score.

### Associations between BMQ scales and with chelation adherence and quality of life

Higher beliefs in the overuse and harm of medicines were correlated with concern about treatments, and higher beliefs in the benefit of medicines were correlated with beliefs in the necessity of treatments (Table 4). Not surprisingly, those who believed their bodies to be more sensitive to medicines (Sensitive Soma) reported higher levels of concern about their treatments, higher beliefs in the overuse and harm of medicines (r=0.24 and 0.23; p<0.001), and lower beliefs in the benefit of medicines (r=−0.17, p<0.001). Those experiencing more side effects felt more concern about their treatments and higher sensitivity to medicines (r=0.13-0.36). Having fewer concerns about treatment was generally associated with higher quality of life (QOL) and fewer symptoms of anxiety and depression. On the other hand, belief in bodily sensitivity to medicines was associated with lower QOL (r=−0.22 - −0.44; p<0.01) and higher symptoms of anxiety and depression (r=0.21, p<0.001 for both).

**Table 4:** Correlations of Beliefs in Medicines Questionnaire (BMQ) scale responses to quality of life and chelation adherence

	**DFO**			**Oral Chelator**			**Iron Burden**	
	**Specific Necessity**	**Specific Concerns**	**Adherence^f^**	**Specific Necessity**	**Specific Concerns**	**Adherence^f^**	**Ferritinx^g^**	**LIC^g^**
General Overuse	0.04 (p=0.66)	**0.29** (p**<0.001**)	−0.09 (p=0.30)	**−0.13** (p=**0.04**)	**0.32** (p**<0.001**)	−0.11 (p=0.10)	0.10 (p=0.06)	**0.13** (p=**0.04**)
General Harm	0.01 (p=0.95)	**0.42** (p**<0.001**)	−0.11 (p=0.23)	**−0.13** (p=**0.03**)	**0.35** (p**<0.001**)	−0.12 (p=0.07)	**0.12** (p=**0.02**)	**0.21** (p**<0.001**)
General Benefit	**0.30** (p**<0.001**)	0.04 (p=0.64)	**0.19** (p=**0.03**)	**0.37** (p**<0.001**)	−0.05 (p=0.46)	−0.002 (p=0.97)	0.02 (p=0.73)	0.03 (p=0.64)
Sensitive Soma	−0.02 (p=0.79)	**0.17** (p=**0.05**)	**−0.23** (p=**0.01**)	−0.09 (p=0.14)	**0.35** (p**<0.001**)	−0.09 (p=0.15)	0.01 (p=0.89)	0.01 (p=0.82)
Coping w/o Treatment^a^	**−0.27** (p=**0.002**)	−0.001 (p=0.98)	0.01 (p=0.95)	**−0.44** (p**<0.001**)	**0.16** (p=**0.01**)	−0.12 (p=0.08)	NA	NA
Side Effects^b^	−0.16 (p=0.06)	**0.50** (p**<0.001**)	−0.11 (p=0.23)	−0.03 (p=0.65)	**0.46** (p**<0.001**)	**−0.14** (p=**0.04**)	NA	NA
QOL Physical – adults^c^	−0.11 (p=0.26)	−0.16 (p=0.11)	NA	−0.06 (p=0.45)	**−0.19** (p=**0.008**)	NA	NA	NA
QOL Mental – adults^c^	0.06 (p=0.55)	−0.10 (p=0.31)	NA	−0.11 (p=0.14)	**−0.23** (p=**0.001**)	NA	NA	NA
QOL Physical – children^d^	0.26 (p=0.19)	−0.04 (p=0.83)	NA	−0.01 (p=0.95)	**−0.34** (p=**0.006**)	NA	NA	NA
QOL Mental – children^d^	0.04 (p=0.85)	−**0.49** (p=**0.01**)	NA	0.09 (p=0.50)	**−0.38** (p=**0.002**)	NA	NA	NA
Anxiety^e^	0.02 (p=0.82)	0.19 (p=0.06)	NA	0.04 (p=0.58)	**0.29** (p**<0.001**)	NA	NA	NA
Depression^e^	0.07 (p=0.46)	**0.34** (p**<0.001**)	NA	0.04 (p=0.59)	**0.28** (p**<0.001**)	NA	NA	NA
Chelation Adherence^f^	−0.10 (p=0.28)	−0.01 (p=0.91)	NA	0.03 (p=0.63)	−0.09 (p=0.18)	NA	NA	NA
Ferritin^g^	0.12 (p=0.17)	**−0.21**^h^ (p=**0.02**)	**−0.28** (p=**0.002**)	−0.06 (p=0.39)	**0.14** (p=**0.03**)	**−0.25** (p**<0.001**)	NA	NA
LIC^g^	−0.04 (p=0.66)	−0.06 (p=0.56)	−0.09 (p=0.39)	−0.04 (p=0.57)	0.14 (p=0.06)	**−0.22** (p=**0.004**)	NA	NA

^a^ single item asking how well patients can cope without their chelation (1–5 scale).

^b^ single item asking about unpleasant side effects of chelation (1–5 scale).

^c^ Physical and Mental Component Summary scales of the SF-36v2 quality of life (QOL) health survey, completed by participants aged 14 and older. Higher scores indicate higher QOL.

^d^ Physical Summary and Psychosocial Summary scales of the PF-28 child health questionnaire (CHQ) , completed by parents/guardians of participants <14 years of age. Higher scores indicate higher QOL.

^e^ Anxiety and depressions scales of the Hospital Anxiety and Depression Scale (HADS), completed by participants aged 14 and older. Higher scores indicate increased anxiety/depression.

^f^ Self-reported (or parent-reported) percent of prescribed doses taken in the last 4 weeks.

^g^ Serum ferritin and Liver iron concentration (LIC) averaged over past year, log-transformed due to skew.

^h^ not significant in adults (r=−0.10, p=0.38).

Average self-reported adherence was 92.2% to DFO and 95.5% to oral chelation (95.6% deferasirox, 94.5% deferiprone), with 76.1% and 87.8% reporting at least 90% adherence, respectively. Surprisingly, adherence to chelation was not associated with beliefs in the necessity of nor concerns about chelation, for either DFO or oral chelation (Table 4). There were also no such trends when analysis was restricted to adults. Adherence was also not associated with the necessity-concerns difference score, a measure of the patients’ cost-benefit analysis (r=−0.05, p=0.57 for DFO; r=0.08, p=0.21 for oral chelation; similar correlations for adults), and did not vary by high/low necessity or concerns or negative/non-negative necessity-concerns difference. On the other hand, average ferritin and LIC over the past year positively correlated with concerns about oral chelation (Table 4), and negatively with the necessity-concerns difference score (r=−0.13, p=0.04 for ferritin; r=−0.13, p=0.09 for LIC). High ferritin (≥2500) was found in 24% of patients reporting high necessity of oral chelation compared to 41% reporting low necessity (p=0.048) and 39% reporting high concerns vs. 23% reporting low concerns (p=0.03). Self-reported adherence negatively correlated with perceived bodily sensitivity to DFO (Sensitive Soma) and with side effects from oral chelation (Table 4).

### Changes in BMQ scores over time

BMQ scores were generally stable over time, with average differences from baseline within one point in either direction for all scales. The only significant change over time was a small decrease in reported concerns about DFO (slope = −0.72, p=0.006), which was no longer significant if analysis was restricted to those participants who remained on DFO (slope = −0.47, p=0.20). Among participants who switched from DFO to oral chelation during TLC follow-up (N=40), concerns about their chelator decreased by 1.76 points (p=0.02). There was no trend towards change in BMQ for those who switched from oral chelation to DFO (N=13).

## Discussion

In this first study to look at beliefs about medication in thalassemia, most participants viewed their thalassemia treatment of transfusion and chelation as very necessary. As expected, their views about concerns were less uniform; having concerns about extensive and invasive treatment can be normal. Nonetheless, these patients generally reported higher beliefs in necessity and lower concerns about their treatment when compared to populations with other diseases [1-3]. Necessity of chelation was similar to that reported for disease-specific medications by diabetic patients [1] and higher than those of patients with asthma, renal disease, cardiac disease, cancer, psychiatric illness, and rheumatoid arthritis [1-3]. Concerns about DFO chelation were similar to concerns about medications in diabetic [1] and oncology patients [2], and lower than all other diseases reported [1-3]; concerns about oral chelation were the lowest of any disease population [1-3]. As blood transfusions are life-sustaining for patients with the more severe forms of thalassemia, it is perhaps not surprising that they view transfusion as more necessary than chelation. Patients immediately feel their response to transfusion with improvement in physical symptoms (e.g., asthenia, back pain). This is in contrast to iron overload which leads to insidious complications without overt symptoms for years. Nevertheless, the high ratings of necessity of chelation are a positive indication that patients understand the benefit of chelation and/or the health consequences of not chelating. This is important because providers do make the effort to educate patients about the necessity of chelation.

### Views about chelation

It is encouraging that regularly transfused patients and those with more complications are especially aware of the necessity of chelation. The higher concerns about DFO compared to oral chelation likely reflects the method of administration of DFO and its disruptiveness to their life. The higher reported sensitivity to medicines of those on DFO compared to oral chelation might also reflect greater disruption DFO has on patient daily life. Indeed, patients who switched from DFO to oral chelation reported decreased concerns, despite the fact that BMQ scores on all scales were generally stable over time. This is consistent with a previous study showing that BMQ General scores remained stable over almost four years, even among those who reported a change in health status [23]. As no measure of the perceived severity of their disease was collected, we were not able to specifically assess how this may have influenced beliefs over time, although a switch from oral chelation to DFO is often precipitated by increased disease severity, and was not associated with any change in beliefs.

Parents expressed significantly higher levels of both necessity and concern compared to children, and necessity and concern beliefs increased with age. This most likely reflects maturation and the attendant worry about long-term effects. Gender, racial, and international differences are not well understood, but may be reflective of the general population rather than thalassemia specific. It is concerning that patients on combination chelation therapy express lower necessity towards both chelators, though this may be expected as need for combination chelation is sometimes due to iron overload resulting from prior non-adherence to monotherapy. Alternatively, combined outcome may be more important to the patient, which may not be reflected by separate beliefs.

### Association with adherence

Correlations between adherence to treatment and the Specific-Necessity and Specific-Concerns scales, along with their difference (cost-benefit analysis) have been reported in diverse diseases and countries and across multiple measures of adherence [1-14,17,18]. This association has been found in a range of countries including the US, UK, Germany, Italy, Sweden, Norway, the Netherlands, and Australia. Methods of quantifying adherence have included self report, the Reported Adherence to Medication (RAM) scale, the Medication Adherence Report Scale (MARS), the Medication Adherence Survey (MAS), pharmacy refill records, serum concentrations of medication, and urinary drug excretion measurements. Only one other study of Swedish migraineurs [15] failed to find an association between necessity/concerns and adherence. This makes the finding of a lack of association between necessity/concerns and self-reported adherence in thalassemia an important finding for the study of the adherence pathway.

Our data shows that thalassemia patients fairly universally understand the necessity of their treatments and have few concerns compared to other patient populations. Unlike other diseases, thalassemia patients have had routine, regular experiences with their treatment throughout their lives. Given the history of poor adherence in the 1970’s and 1980’s, providers spend a lot of energy educating patients about the importance of chelation adherence. These data show that this effort pays off with high necessity-concern scores. What is concerning is this knowledge does not appear to translate into adherence action.

Clearly adherence is a complex problem. In a recent publication, we reported high chelation adherence in this thalassemia population, with an apparent surge in self-reported adherence rates with the recent introduction of alternative choices of oral chelators [24]. However, this study used adherence self-reports that may overestimate actual adherence. Using clinical assessments such as serum ferritin and LIC may better reflect past or present chelation adherence, but these measures of iron burden may also reflect inadequate dosing. Complicating the picture is the observed positive association between concerns with oral chelation and ferritin and LIC that suggests an association of adherence with concerns. There is also the negative correlation between concerns with DFO and ferritin that is difficult to interpret, although anecdotal evidence suggests that patients who choose to use DFO tend to be older, while younger patients have to use DFO because of poor compliance.

Overall, problems with administration and side effects appeared to be common causes of poor chelation adherence [24]. Although side effects are strongly correlated with the Concerns scale, it appears that side effects themselves may be the better predictor of adherence, as evidenced by the association between ferritin, LIC, and non-adherence to oral chelation with side effects. Thus it appears that thalassemia patients may not base adherence on a health cost-benefit analysis of the necessity of chelation, especially with DFO, but rather recognize that chelation is absolutely necessary but sometimes difficult to maintain.

Consistent with a previous study [16], adherence was found to negatively correlate with perceived personal sensitivity to medications (Sensitive Soma). Addressing patients’ beliefs about the tolerability of medications has the prospect for gains not just in adherence, but also in quality of life and decreased anxiety/depression, which have been found to be impaired in thalassemia [25,26]. How to address these beliefs is a challenge because patient experience of side effects and clinical complications of thalassemia are not only associated with higher sensitive Soma scores but also impaired quality of life [25].The BMQ data clearly show that thalassemia patients on both types of chelators express an understanding of the need for the drug, but then may simply not use it to the extent prescribed. This suggests that the tried response that appears to work for many diseases - more education – is unlikely to be adequate. What is clear is that patient beliefs are out of step with their practices. Given this, the logical intervention would be to deploy strategies that help patients align their beliefs with their practices. Anecdotal, unpublished data from the University College London Thalassemia Program appear to show that cognitive-behavioral therapy does improve adherence and long-term outcome in thalassemia. While the costs of this behavioral intervention can appear to be immediately expensive, the improved long-term outcome and reduced need for clinical interventions because of complications due to iron-overload far outweigh this burden.

## Conclusions

One goal of deploying multiple “quality of life” instruments in the TLC was to measure known social and behavioral factors that influence patient adherence behaviors in order to inform intervention. The data from the deployed set of instruments including the BMQ and Sensitive Soma clearly reinforce the reality that adherence is a complex and multifaceted problem. This study has many strengths and is the first study of beliefs in medications in thalassemia. We were able to analyze a large, racially diverse, international sample of thalassemia patients, almost all on transfusion therapy, mostly using deferoxamine or deferasirox for chelation. This study also had the advantage of descriptively comparing beliefs to other disease populations, as well as investigating associations with QOL, symptoms of anxiety and depression, and adherence. Finally, the longitudinal cohort allowed for exploration of changes in beliefs over time, especially for patients who switched chelators during follow-up. This study also had several limitations that may impact its generalizability. First, less adherent patients may also be less likely to participate in research, and beliefs in medicine may influence decisions to attend study visits, thereby potentially skewing the resulting data towards higher adherence and more positive beliefs in medicine. Second, adherence was measured from patient report, which is likely overestimated. Additionally, the SF-36v2 health survey has only been formally validated in adults, but we chose to use the instrument on participants aged 14–17 as well, in order to assess the adolescent view. Quality of life in children was measured solely by parent report; additional measurement by child report would be instructive. Finally, no information on specific side effects or adherence to transfusion therapy was collected. As non-adherence to transfusion therapy results in lower hemoglobin levels, which generally immediately affects how patients feel, it would be of interest to measure. Additionally, comparison of the beliefs of thalassemia patients to those with other hematological diseases would be worthwhile.

Clinically, these findings are instructive. Data suggests that patients do know about the importance of their medications. Providers do need to continue to reinforce the necessity of the clinical regimens, and manage patient concerns with them. They also need to recognize that patient adherence is not simply predicated on patients being better educated, but also learning how to use that knowledge in the face of other life complications. This assessment of individual beliefs regarding thalassemia treatment can help health providers better motivate their patients. While change in general beliefs may be hard to implement, perceived sensitivity to chelation may represent an opportunity for targeted intervention. Thalassemia patients may benefit from perceiving that their bodies can actually tolerate the prescribed therapy, and continued efforts to find effective chelators that are painless and without significant side effects seem warranted. In the meantime, devising strategies and interventions to help patients effectively cope with side effects might be ultimately beneficial in lowering body iron burden. As beliefs were found to differ by age, sex, and race, support groups mixing patients could be of interest. Despite their requirement for demanding, multimodal therapy, it is encouraging that thalassemia patients have positive views about medicine, with high belief in their necessity and relatively low concerns about their therapy.

## Appendix 1

### Beliefs about Medicines Questionnaire (BMQ) Items

*Rated: 1=strongly disagree, disagree, uncertain, agree, strongly agree=5Scoring: sum of responses to individual itemsHigher scores indicate higher agreement with the scale items*

### Specific-Necessity: (5–25 points)

My health, at present, depends on my medicines.My life would be impossible without my medicines.Without my medicines I would be very ill.My health in the future will depend on my medicines.My medicines protect me from becoming worse.

### Specific-Concerns: (5–25 points)

Having to take medicines worries me.I sometimes worry about long-term effects of my medicines.My medicines are a mystery to me.My medicines disrupt my life.I sometimes worry about becoming too dependent on my medicines.

### General-Overuse: (4–20 points)

Doctors use too many medicines.Natural remedies are safer than medicines.Doctors place too much trust on medicines.If doctors had more time with patients they would prescribe fewer medicines.

### General-Harm: (4–20 points)

People who take medicines should stop their treatment for a while every now and again.Most medicines are addictive.Most medicines are poisons.Medicines do more harm than good.

### General-Benefit: (4–20 points)

Medicines help many people to live better lives.In most cases the benefits of medicines outweigh the risks.In the future medicines will be developed to cure most diseases.Medicines help many people to live longer.

### Sensitive-Soma: (5–25 points)

My body is very sensitive to medicines.My body over-reacts to medicines.I usually have stronger reactions to medicines than most people.I have had a bad reaction to medicines in the past.Even very small amounts of medicine can upset my body.

## Appendix 2

The following institutions and researchers contributed to the Thalassemia Clinical Research Network Thalassemia Longitudinal Cohort data reported in this paper.Children’s Hospital, Boston: Ellis Neufeld, MD, PhD, Principal Investigator, Jennifer Braunstein, NP, Research Nurse, Amber Smith, Study Coordinator, Latoya Lashley, Study Coordinator; Satellite: University of Texas Southwestern Medical Center at Dallas, Charles Quinn, MD, MS, Principal Investigator, Deborah Boger, RN, MSN, PNP, Study Coordinator, Leah Adix, Study Coordinator, Sandra Richardson, Study Coordinator; Children's Healthcare of Atlanta, Jeanne Boudreaux, MD, Principal Investigator, Leann Hassen, Study Coordinator; Baylor College of Medicine, Brigitta Mueller, MD, Principal Investigator, Bogden Dino, Study Coordinator. Weill Medical College of Cornell University: Patricia Giardina, MD, Principal Investigator, Dorothy Kleinert, RN, Research Nurse; Satellite: Winthrop University Hospital, Mark Weinblatt, MD, Principal Investigator, Linda Skelly, Study Coordinator. The Children’s Hospital of Philadelphia: Janet Kwiatkowski, MD, Principal Investigator, Marie Martin, RN, Research Nurse, Sage Green, Study Coordinator; Satellite: Children’s Memorial Hospital, Chicago, IL, Alexis Thompson, MD, Principal Investigator, Janice Beatty, RN, Research Nurse, Diane Calamaras, RN, CPNP, Research Nurse, Pauline Hess, study coordinator. Children’s Hospital at Oakland: Elliott Vichinsky, MD, Principal Investigator, Dru Foote, NP, Research Nurse, Nancy Sweeters, Study Coordinator, Olivia Vega, Study Coordinator; Satellites: Children’s Hospital of Los Angeles, Thomas Coates, MD, Principal Investigator, Susan Carson, RN, Research Nurse, Eun Ha Pang, Study Coordinator, Rachna Khanna, Study Coordinator; Stanford Hospital, Michael Jeng, MD, Principal Investigator, Kokil Bakshi, Clinical Research Associate; Children's and Women's Health Center of British Columbia, John Wu, Principal Investigator, Heather McCartney, RN, Research Nurse, Colleen Fitzgerald, Study Coordinator, Stephanie Badour, Study Coordinator. Toronto General Hospital, Toronto, Ontario, Canada: Nancy F. Olivieri, MD, Principal Investigator, Vivek Thayalasuthan, Study Coordinator; Satellite: Hospital for Sick Children, Isaac Odame, MD, Principal Investigator, Manuela Merelles-Pulcini, RN, Study Coordinator. University College London, John Porter, MD, Principal Investigator, Cindy Bhagwandin, Study Coordinator; Satellite: Whittington Hospital, Farrukh Shah, MD, Principal Investigator. NHLBI oversight, Kathryn Hassell, MD. Data Coordinating Center: New England Research Institutes, Sonja McKinlay, PhD, Principal Investigator, Lisa Virzi, RN, MS, MBA, Project Director, Felicia Trachtenberg, PhD, Senior Statistician.

## Abbreviations

TCRN: Thalassemia Clinical Research Network; BMQ: Beliefs in Medicine Questionnaire; TLC: Thalassemia Longitudinal Cohort; DFO: Deferoxamine; SSAS: Sensitive Soma Assessment Scale; HADS: Hospital Anxiety and Depression Scale; QOL: Quality of Life; CHQ: Child Health Questionnaire; LIC: Liver Iron Concentration; ANOVA: Analysis of Variance; RAM scale: Reported Adherence to Medication; MARS: Medication Adherence Report Scale; MAS: Medication Adherence Survey.

## Competing interests

The following authors declare the following financial conflicts of interest. All other authors report no conflicts of interest. Ellis Neufeld: research funding from Novartis and Ferrokin Biosciences. Charles Quinn: advisory board member of ApoPharma.

## Authors’ contributions

FT performed the statistical analysis and drafted the manuscript. LM, ZP, RG, and AS helped with interpretation of data and manuscript revision. JK and EN participated in study design, acquisition of data, and manuscript revision. DH participated in acquisition of data, interpretation of data, and manuscript revision. AT, CQ, and NO participated in acquisition of data and manuscript revision. RH designed the BMQ, helped with interpretation of data, and manuscript revision. RY participated in study design, interpretation of data, and helped draft the manuscript. All authors read and approved the final manuscript.

## Authors’ information

FT is senior statistician for the TCRN. LM, AS, and RY are TCRN consultants with expertise in quality of life and anxiety/depression. JK, EN, ZP, AT, CQ, RG, and NO are hematologists at participating TCRN sites. DH is a hematologist nurse at a participating TCRN site. RH is the developer of the BMQ scales.

### For the Thalassemia Clinical Research Network

This is publication number 22 of the Thalassemia Clinical Research Network (TCRN). A list of TCRN member institutions and staff appears in Appendix 2.
